# Clinical Effectiveness of Regdanvimab Treatment for Mild-to-Moderate COVID-19: A Retrospective Cohort Study

**DOI:** 10.1016/j.curtheres.2022.100675

**Published:** 2022-05-16

**Authors:** Young Rock Jang, Yoon Ju Oh, Jin Yong Kim

**Affiliations:** 1 Division of Infectious Diseases, Department of Internal Medicine, Incheon Medical Center, Incheon, Republic of Korea; 2 Division of Metabolism and Endocrinology, Department of Internal Medicine, Incheon Medical Center, Incheon, Republic of Korea

**Keywords:** COVID-19, CT-P59, regdanvimab, retrospective study, SARS-CoV-2

## Abstract

**Background:**

In a Phase III study, regdanvimab (CT-P59) reduced the risk of hospitalization or death versus placebo in patients with mild-to-moderate coronavirus disease 2019 (COVID-19).

**Purpose:**

We performed a retrospective cohort study of patients with COVID-19 to examine the effect of regdanvimab versus standard of care (SoC) on oxygen saturation.

**Methods:**

We reviewed patients with mild-to-moderate COVID-19 confirmed by reverse transcription-polymerase chain reaction at a single hospital in the Republic of Korea. The primary efficacy end point was the proportion of patients deteriorating with peripheral capillary oxygen saturation <94% on room air up to day 28.

**Results:**

A total of 127 patients were treated for COVID-19 with regdanvimab, 190 with SoC. The proportion of patients deteriorating with peripheral capillary oxygen saturation <94% on room air up to day 28 was 13.4% with regdanvimab and 39.5% with SoC (*P <* 0.0001); median time (range) until sustained recovery of fever was 2.0 (0.2–14.8) and 4.2 (0.1–17.1) days, respectively. Supplemental oxygen was required by 23.6% of patients with regdanvimab and 52.1% with SoC (*P<*0.0001) for a mean of 6.3 and 8.7 days, respectively (*P =* 0.0113); no patients needed mechanical ventilation. Compared with SoC, hospitalization was shorter with regdanvimab (mean = 11.1 vs 13.6 days; 63.8% vs 31.6% discharged within 11 days; both *P* values *<* 0.0001). Fewer regdanvimab-treated patients required remdesivir (14.2% vs 43.2%; *P <* 0.0001). There were no deaths. Two patients had adverse reactions with regdanvimab.

**Conclusions:**

This real-world study indicates that regdanvimab can prevent deterioration in patients with mild-to-moderate COVID-19. (*Curr Ther Res Clin Exp*. 2022; 83:XXX–XXX)

## Introduction

Since December 2019, the global spread of the novel, severe acute respiratory syndrome coronavirus 2 (SARS-CoV-2), which causes coronavirus disease 2019 (COVID-19), has been rapid and relentless. According to the World Health Organization (WHO), more than 383 million confirmed cases of COVID-19 have been recorded worldwide as of February 3, 2022, resulting in more than 5.6 million deaths.^1^ A global pandemic of this unprecedented scale requires a worldwide immunization campaign if spread is to be curtailed. Alongside the challenge of producing effective and safe vaccines on a global scale, there is a need to ensure their affordable cost, fair allocation, efficient deployment, and public acceptance.^2^

Notwithstanding the hurdles involved in ensuring success of the rapid international immunization response, there also remains a need for effective new treatments for patients with COVID-19.^3^^,^^4^ Although vaccines against COVID-19 have been developed and many people have subsequently been vaccinated,^1^ new effective pharmacologic treatments for patients who do become infected remain key to improving outcomes, to reduce the burden on both patients and health care systems.^4^

Since the pandemic began, hundreds of COVID-19 clinical trials have evaluated the therapeutic efficacy of new and repurposed treatments, including antiviral agents that aim to block SARS-CoV-2 activity, anti-inflammatory medicines to reduce the resultant immune response, and antibody therapies to assist the body's immune response to fight the virus.^3^^,^^4^ To date, only a handful of treatments have been deemed to be definitively effective.^4^ The significant potential for the use of monoclonal antibodies (mAbs) in the treatment of emerging infectious diseases has been noted,^5^ and during the COVID-19 pandemic, mAbs designed to disrupt viral cell entry and reduce infectivity by targeting the SARS-CoV-2 spike protein have been evaluated in various clinical settings, including prophylaxis and in patient populations with early or late disease.^6^ Positive outcomes, including reduced viral load and lower hospitalization rates, have been seen in patients with COVID-19.^6^, ^7^, ^8^, ^9^

The SARS-CoV-2 mAb, regdanvimab (CT-P59), which blocks interaction between the SARS-CoV-2 spike-protein receptor binding domain and the angiotensin-converting enzyme-2 receptor, demonstrated a promising safety profile in Phase I studies, showing potential antiviral and clinical efficacy in patients with mild symptoms of COVID-19,^10^ and was subsequently evaluated in a Phase II/III randomized, placebo-controlled, double-blind study. Preliminary findings from the Phase III part of the study demonstrated that regdanvimab significantly reduced the likelihood of requiring hospitalization or oxygen therapy, or of experiencing mortality due to COVID-19 over 28 days by 72%, when compared with placebo in patients with mild-to-moderate COVID-19 at high risk of progressing to severe COVID-19, and by 70% for all patients (both *P* values *<* 0.0001).^11^^,^^12^ Following conditional marketing authorization for regdanvimab, issued by the Korean Ministry of Food and Drug Safety in February 2021, full regulatory approval with an expansion of indication and reduced infusion duration was granted on September 17, 2021.^13^ Emergency Use Authorization was granted in Brazil on August 11, 2021.^14^ In November 2021, regdanvimab was recommended for marketing authorization by the European Medicines Agency Committee for Medicinal Products for Human Use for treatment of confirmed COVID-19 in adult patients at high risk of progressing to severe disease.^15^ On December 6, 2021, the Therapeutic Goods Administration granted provisional approval for the use of regdanvimab in Australia.^16^

As of February 4, 2022, 44,413 patients with COVID-19 had received treatment with regdanvimab at 279 hospitals in the Republic of Korea.^17^ To further our understanding of the effectiveness of regdanvimab, we conducted a retrospective cohort study in patients with mild-to-moderate COVID-19 undergoing treatment with regdanvimab or standard of care (SoC) (ie, antipyretics, analgesics, and antibiotics, administered according to clinical need), examining effects of treatment on clinical outcome, and herein report the 28-day results after hospitalization.

Low oxygen saturation levels are a known risk factor for deterioration and mortality among patients with COVID-19,^18^^,^^19^ with dangerously low oxygen levels sometimes occurring without associated clinical symptoms (silent hypoxemia). Typically, these patients have a very poor outcome. In light of this, we examined the effect of regdanvimab on oxygen saturation versus SoC.

## Participants and Methods

### Study design

This was a single-center, retrospective cohort study of patients with COVID-19 (confirmed by reverse transcription-polymerase chain reaction [RT-PCR]) and mild or moderate associated COVID-19 symptoms at Incheon Medical Center, Incheon, in the Republic of Korea. According to the treatment and management policy in the Republic of Korea, patients with mild cases of COVID-19 are admitted to residential treatment centers for monitoring and medical treatment, whereas patients with moderate, severe, or extremely severe cases of COVID-19 receive in-hospital treatment at infectious disease hospitals or nationally designated treatment facilities, depending on the severity of their condition.^20^ In this study, disease severity was classified as defined by the WHO.^21^ The protocol was reviewed and approved by local institutional review boards on August 2, 2021, before study initiation. For this retrospective analysis, formal informed consent was not required in accordance with Article 16 of the Bioethics and Safety Act of the Republic of Korea.^22^

The primary objective of the study was to evaluate the clinical efficacy of regdanvimab in comparison with that of nonregdanvimab treatment, as determined by the proportion of patients deteriorating with peripheral capillary oxygen saturation (SpO_2_) <94% on room air up to day 28. Secondary objectives were to evaluate additional efficacy end points and the safety profile of regdanvimab.

Medical records of all patients admitted to the center with COVID-19 from September 2020 to July 2021 were retrospectively reviewed, and patients treated with regdanvimab or other treatments were assessed for eligibility. Anonymized data from medical records (including electronic medical records) were collated in electronic case report forms for statistical analysis.

The standard administration of regdanvimab at Incheon Medical Center was a dose of 40 mg/kg as an intravenous infusion over 90 minutes (±15 minutes), not later than 7 days after symptom onset. Patients in the nonregdanvimab cohort received SoC.

### Study population

Adults aged ≥18 years with a confirmed first diagnosis of COVID-19 by RT-PCR with oxygen saturation of >94% on room air, not requiring supplemental oxygen, were eligible for inclusion in the study if they had ≥1 mild or moderate COVID-19–associated symptoms (including, but not limited to fever [ie, body temperature ≥38°C], shortness of breath, cough, diarrhea, sputum, sore throat, headache, myalgia, and loss of taste or smell) and were at high risk of progression to severe COVID-19. Factors determining high-risk status were those according to the indication in the conditional approval for regdanvimab granted by the Korean Ministry of Food and Drug Safety.^13^ Patients were excluded if they had a previous diagnosis of COVID-19 or experienced severe COVID-19–related conditions within 7 days before receiving any treatments for COVID-19 (in the opinion of the investigator), if they were ineligible for treatment with regdanvimab, if they had participated in clinical studies of any other investigational medical products for the treatment of COVID-19 (including but not limited to convalescent plasma, remdesivir, and hydroxychloroquine), or if they were considered unsuitable for participation at the investigator's discretion. Patients who had received SARS-CoV-2 vaccination were not eligible for inclusion.

### Study end points

The primary efficacy end point was the proportion of patients deteriorating with SpO_2_ <94% on room air up to day 28. Secondary efficacy end points included the time until sustained recovery of fever due to COVID-19, where sustained recovery of fever was defined as body temperature maintained <38°C; the proportion of patients requiring supplemental oxygen due to COVID-19 up to day 28 and the duration of supplemental oxygen therapy; and the proportion of patients requiring mechanical ventilation due to COVID-19 up to day 28. Other secondary outcomes were the proportion of patients deteriorating with SpO_2_ <90% on room air up to day 28, the duration of hospitalization due to COVID-19 (excluding patients transferred to other hospitals during the hospitalization period), the proportions of patients discharged up to day 11 and day 14, the proportions of patients requiring remdesivir or corticosteroids due to COVID-19 up to day 28, and 28-day all-cause mortality. At the time of study, in the Republic of Korea remdesivir was recommended for hospitalized patients with severe COVID-19 who had either SpO_2_ <94% in room air, or required supplementary oxygen therapy, or required mechanical ventilation or extracorporeal membrane oxygenation. In this context, the proportion of patients receiving remdesivir may therefore represent the proportion of patients progressing to severe disease, corresponding to the indication of remdesivir. A post hoc analysis of the primary and secondary efficacy end points was performed in patients aged ≥60 years. The authors note that the prescription of remdesivir was originally included in the first analysis of the primary end point. This was appropriate for a single-center study in the Republic of Korea; however, the prescribing criteria for remdesivir differ from country to country. Different prescription standards had the potential to cause confusion when interpreting the results, so it was considered appropriate to focus only on the objective and widely applicable SpO_2_ measurement.

For the purpose of evaluation, Day 1 for patients in both groups was defined as the hospitalization date, and the date upon which any treatment was started. Because SoC includes treatments intended to alleviate symptoms (eg, antipyretics, analgesics, and antibiotics) these were administered as required during hospitalization according to the clinical status of the patient, at the discretion of the investigator.

The safety profile was evaluated up to day 28 by assessing adverse events (AEs) related to the administration of regdanvimab.

### Statistical analysis

A sample size of approximately 400 patients was proposed based on the estimated number of admissions of patients with COVID-19 to the study center during the data collection period, rather than a formal statistical hypothesis. All efficacy end points were analyzed in the efficacy set (ie, all patients who had received a full dose of regdanvimab or who had been admitted for the treatment of COVID-19 and had ≥1 postadmission evaluation for efficacy).

The primary efficacy end point was presented along with the 95% Wilson score CI for the proportion in each group and tested at the 2-sided significance level using Fisher exact test. A *P* value < 0.05 was determined to indicate statistical significance. The difference of proportions between the regdanvimab and nonregdanvimab groups was also calculated along with the 95% CI derived by Farrington-Manning exact test.

Secondary efficacy end points were summarized according to regdanvimab and nonregdanvimab groups by means of descriptive statistics or frequency tables. The χ^2^ test was used for categorical variables and the Student *t* test for continuous variables. *P* values were presented for comparison between the 2 groups with no adjustments for multiple testing.

Safety analyses were performed on the safety set (ie, all patients who had received a full or partial dose of regdanvimab or who had been admitted for the treatment of COVID-19), unless otherwise indicated, with AEs coded by System Organ Class and Preferred Term according to the Medical Dictionary for Regulatory Activities, version 24.0, and graded according to the Common Terminology Criteria for Adverse Events, version 5.0. Prior and concomitant medications were coded by drug class and Preferred Term using the WHO Drug Dictionary, September 2020 version.

All statistical analyses were conducted using Statistical Analysis System software, version 9.4 (SAS Institute Inc, Cary, North Carolina).

## Results

### Patient population and baseline characteristics

**Figure 1:**
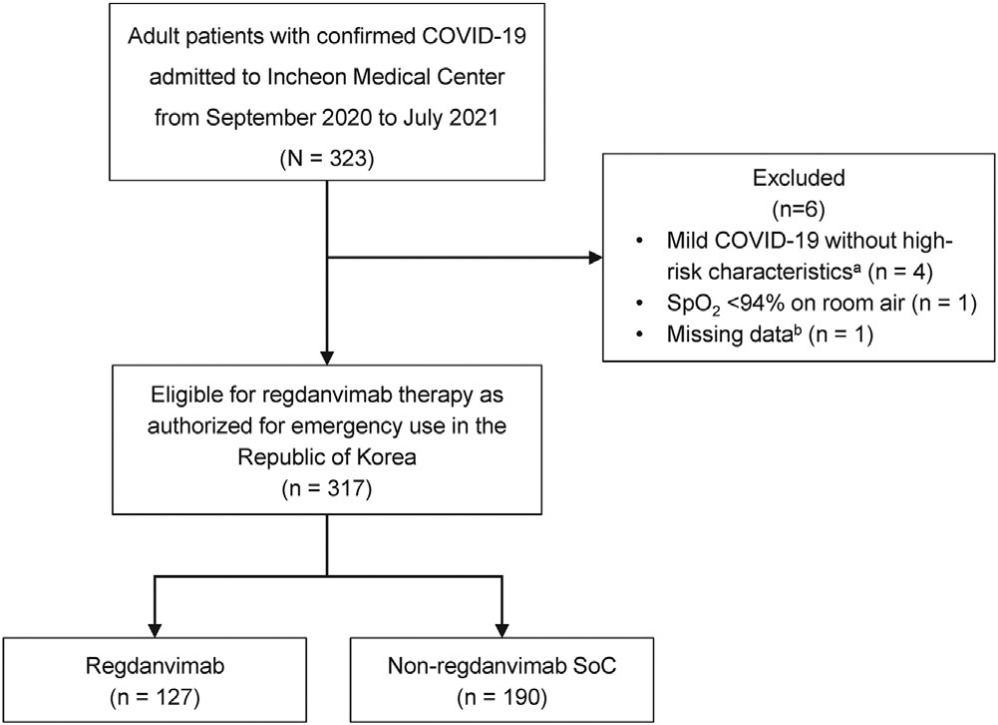
Patient flow diagram. ^a^High-risk patients were defined as patients aged ≥60 years or with underlying conditions (ie, 1 or more of cardiovascular disease, chronic respiratory disease, diabetes, or hypertension). ^b^Records for 1 patient were missing the onset date of coronavirus disease 2019 (COVID-19) symptoms. SoC = standard of care; SpO2 = peripheral capillary oxygen saturation.

A total of 323 patients with RT-PCR–confirmed COVID-19 were admitted with mild-to-moderate COVID-19 at Incheon Medical Center from September 2020 to July 2021, and 317 were deemed eligible for regdanvimab per the Emergency Use Authorization (Figure 1).

All eligible patients before the date when regdanvimab was authorized for emergency use in the Republic of Korea (February 5, 2021) were enrolled in the nonregdanvimab cohort. Subsequently, consecutive patients were assigned to the respective cohorts according to regdanvimab use. In total, 127 patients who had been administered regdanvimab were included in the analysis. An additional 190 patients received other SoC for COVID-19 without regdanvimab (ie, the nonregdanvimab cohort), which largely consisted of palliative treatments for symptom control.

Demographic characteristics, including median age, sex, and body mass index were balanced between the regdanvimab and nonregdanvimab cohorts. The proportion of patients aged ≥60 years was higher in the nonregdanvimab than regdanvimab cohort; the proportions of patients with moderate disease severity and with abnormal vital signs were higher in the regdanvimab cohort. Baseline demographic and disease characteristics are summarized in Table 1. Medical history data for high-risk patients are summarized in Table 2.

**Table 1:** Baseline demographics and characteristics of all eligible patients.

Characteristic	Regdanvimab (n = 127)	Nonregdanvimab (n = 190)	Total (N = 317)
Age, y			
Median (range)	61 (20–90)	64 (23–91)	63 (20–91)
≥60*	73 (57.5)	137 (72.1)	210 (66.2)
Male*	53 (41.7)	79 (41.6)	132 (41.6)
BMI†	24.5 (3.9)	25.0 (3.4)	24.8 (3.6)
Baseline COVID-19 symptoms*			
Fever	60 (47.2)	85 (44.7)	147 (45.7)
Shortness of breath	4 (3.1)	4 (2.1)	8 (2.5)
Cough	62 (48.8)	84 (44.2)	146 (46.1)
Diarrhea	6 (4.7)	6 (3.2)	12 (3.8)
Sputum	28 (22.0)	30 (15.8)	58 (18.3)
Sore throat	38 (29.9)	32 (16.8)	70 (22.1)
Headache	34 (26.8)	37 (19.5)	71 (22.4)
Myalgia	32 (25.2)	48 (25.3)	80 (25.2)
Lack of taste or smell	5 (3.9)	11 (5.8)	16 (5.0)
Severity of COVID-19*			
Mild	15 (11.8)	48 (25.3)	63 (19.9)
Moderate	112 (88.2)	142 (74.7)	254 (80.1)
Pneumonia*	112 (88.2)	142 (74.7)	254 (80.1)
Abnormal vital signs*			
Body temperature ≥38°C	63 (49.6)	68 (35.8)	131 (41.3)
Heart rate >100 beats/min	26 (20.5)	31 (16.3)	57 (18.0)
Respiratory rate ≥20 breaths/min	95 (74.8)	129 (67.9)	224 (70.7)
Baseline SpO_2_ level on room air, %‡	97.0 (92–99)	98.0 (94–100)	97.0 (92–100)

BMI = body mass index; COVID-19 = coronavirus disease 2019; SpO_2_ = peripheral capillary oxygen saturation.

⁎ Values are presented as n (%).

† Values are presented as mean (SD).

‡ Values are presented as median (range).

**Table 2:** Medical history* at baseline.

	Regdanvimab (n = 127)	Nonregdanvimab (n = 190)	Total (N = 317)
Total No. of medical history entries	115	153	268
Patients with ≥1 medical history entry†	85 (66.9)	114 (60.0)	199 (62.8)
Preferred term†			
Angina pectoris	6 (4.7)	2 (1.1)	8 (2.5)
Arrhythmia	6 (4.7)	0	6 (1.9)
Cardiac disorder	9 (7.1)	3 (1.6)	12 (3.8)
Myocardial infarction	0	1 (0.5)	1 (0.3)
Diabetes mellitus	28 (22.0)	59 (31.1)	87 (27.4)
Asthma	0	1 (0.5)	1 (0.3)
Bronchial hyperreactivity	1 (0.8)	0	1 (0.3)
Bronchiectasis	0	1 (0.5)	1 (0.3)
Bronchitis, chronic	1 (0.8)	0	1 (0.3)
Chronic obstructive pulmonary disease	3 (2.4)	7 (3.7)	10 (3.2)
Emphysema	1 (0.8)	0	1 (0.3)
Interstitial lung disease	1 (0.8)	0	1 (0.3)
Pneumothorax	0	1 (0.5)	1 (0.3)
Rhinitis, allergic	2 (1.6)	0	2 (0.6)
Hypertension	56 (44.1)	77 (40.5)	133 (42.0)
Vascular stenosis	0	1 (0.5)	1 (0.3)

⁎ Medical history was summarized for high-risk patients with underlying conditions, as defined in the indication of the conditional marketing authorization for regdanvimab in the Republic of Korea (1 or more of cardiovascular disease, chronic respiratory disease, diabetes, or hypertension).

† Values are presented as n (%).

### Efficacy

**Figure 2:**
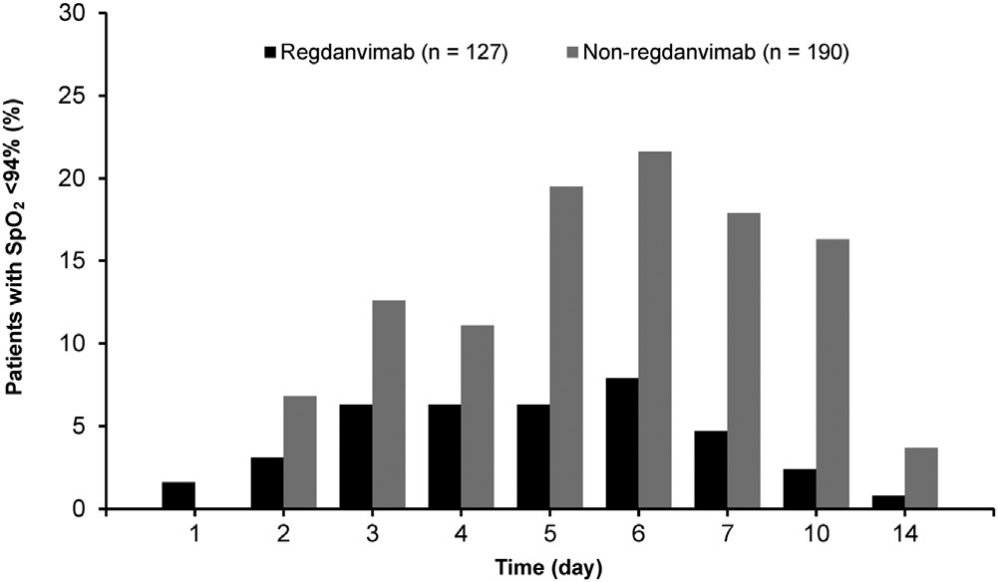
Proportion of patients with peripheral capillary oxygen saturation (SpO_2_) <94% up to day 14 of hospitalization.

The proportion of patients with COVID-19 who deteriorated with SpO_2_ <94% on room air up to day 28 (primary end point) was 13.4% with the regdanvimab cohort and 39.5% with the nonregdanvimab cohort (–26.1%; 95% CI, –35.1 to –15.9; *P <* 0.0001) (Table 3). Among patients in the regdanvimab cohort, the proportion who met the primary end point was similar in the subgroup of patients receiving regdanvimab <3 days after earliest symptom onset (9 out of 54 patients; 16.7%) and the subgroup receiving regdanvimab ≥3 days after earliest symptom onset (8 out of 73 patients; 11.0%; *P =* 0.43). The proportion of patients with SpO_2_ <94% was higher in the nonregdanvimab than regdanvimab cohort across all time points up to day 14 (Figure 2).

**Table 3:** Primary efficacy end point data up to day 28.

	Regdanvimab (n = 127)	Nonregdanvimab (n = 190)	Difference [95% CI]*	*P* value for comparison between cohorts†
Primary analysis				
Patients with SpO_2_ <94% on room air‡ 95% CI§	17 (13.4) 8.5 to 20.4	75 (39.5) 32.8 to 46.6	−26.1 [−35.1 to −15.9]	< 0.0001
Subgroup analysis				
	Duration of symptoms in the regdanvimab cohort||			
	<3 days (n = 54)	≥3 days (n = 73)		
Patients with SpO_2_ <94% on room air‡ 95% CI§	9 (16.7) 9.0 to 28.7	8 (11.0) 5.7 to 20.2	5.7 [−6.7 to 19.6]	0.4319

SpO_2_ = peripheral capillary oxygen saturation.

⁎ Farrington-Manning score exact 95% CI for the proportional difference between cohorts.

† Fisher exact test.

‡ Values are presented as n (%).

§ Wilson 95% CI for each proportion.

|| Time (days) since the earliest symptom was calculated as (date of regdanvimab administration − date of earliest symptom start) in the regdanvimab cohort.

Secondary efficacy end point data are summarized in Table 4. Patients who received regdanvimab recovered more quickly from fever than those who did not receive regdanvimab; the mean (SD) time until sustained recovery of fever (body temperature maintained <38°C) was 2.9 (3.0) days and 4.6 (3.3) days, respectively (*P <* 0.0001). The proportion of patients with SpO_2_ <90% on room air up to day 28 (ie, severe hypoxemia) was 3.1% in the regdanvimab cohort and 11.1% in the nonregdanvimab cohort (*P =* 0.0105). Supplemental oxygen was required by 23.6% of patients in the regdanvimab cohort in comparison with 52.1% in the nonregdanvimab cohort (*P <* 0.0001), for a mean (SD) duration of 6.3 (3.6) and 8.7 (4.7) days, respectively (*P =* 0.0113). No patients in either cohort needed mechanical ventilation. The mean (SD) duration of hospitalization was shorter in the regdanvimab than nonregdanvimab cohort: 11.1 (3.6) versus 13.6 (4.1) days (*P <* 0.0001). The proportion of patients who were discharged up to day 11 was higher in the regdanvimab than nonregdanvimab cohort (63.8% vs 31.6%; *P <* 0.0001). A higher proportion of patients in the nonregdanvimab cohort (43.2%) than the regdanvimab cohort (14.2%) required remdesivir therapy (*P <* 0.0001). Similar proportions of patients in the regdanvimab and nonregdanvimab cohorts received corticosteroid therapy due to COVID-19 (5.5% vs 6.3%, respectively; *P =* 0.768). No patients in either cohort died during the study follow-up period.

**Table 4:** Secondary efficacy end point data up to day 28.

	Regdanvimab (n = 127)	Nonregdanvimab (n = 190)	*P* value* for comparison between cohorts
Time to sustained recovery of fever† Days‡	102 2.9 (3.0)	134 4.6 (3.3)	< 0.0001
Patients with SpO_2_ <90% on room air§	4 (3.1)	21 (11.1)	0.0105
Patients requiring supplemental oxygen§	30 (23.6)	99 (52.1)	< 0.0001
Duration of supplemental oxygen therapy, d‡	6.3 (3.6)	8.7 (4.7)	0.0113
Patients requiring mechanical ventilation§	0	0	–
Duration of hospitalization due to COVID-19 Days‡	127 11.1 (3.6)	189 13.6 (4.1)	< 0.0001
Patients discharged up to day 11§	81 (63.8)	60 (31.6)	< 0.0001
Patients discharged up to day 14§	110 (86.6)	133 (70.0)	0.0006
Patients requiring remdesivir therapy§	18 (14.2)	82 (43.2)	< 0.0001
Patients requiring corticosteroid therapy§§	7 (5.5)	12 (6.3)	0.768
Patients with all-cause mortality§	0	0	–

COVID-19 = coronavirus disease 2019; SpO_2_ = peripheral capillary oxygen saturation.

⁎ The χ^2^ test for categorical variables and Student *t* test for continuous variables.

† Defined as body temperature maintained <38°C. Fever recovery duration was defined as [(first fever recovery date/time after last body temperature ≥38°C) – (first date/time of body temperature ≥38°C)].

‡ Values are presented as mean (SD).

§ Values are presented as n (%).

In the post hoc analysis of patients aged ≥60 years, the proportion of patients who deteriorated with SpO_2_ <94% on room air up to day 28 was 15.1% in the regdanvimab cohort and 45.3% in the nonregdanvimab cohort (*P <* 0.0001) (Table 5). For all evaluated secondary efficacy end points, other than the duration of supplemental oxygen therapy and the proportion of patients requiring corticosteroid therapy, there was a statistically significant difference between cohorts (Table 5).

**Table 5:** Primary and secondary efficacy end point data up to day 28, in patients aged 60 years or older.

	Regdanvimab (n = 73)	Nonregdanvimab (n = 137)	*P* value for comparison between cohorts*^,^†
Primary end point			
Patients with SpO_2_ <94% on room air‡	11 (15.1) [8.6 to 25.0]	62 (45.3) [37.2 to 53.6]	< 0.0001
Difference§	−30.2 [−41.4 to −16.5]		
Secondary end points			
Time to sustained recovery of fever||	55	96	0.0070
Days¶	3.3 (3.6)	4.9 (3.3)	
Patients requiring supplemental oxygen#	17 (23.3)	79 (57.7)	< 0.0001
Duration of supplemental oxygen therapy, d¶	7.0 (4.2)	9.0 (4.9)	0.1218
Patients requiring mechanical ventilation#	0	0	−
Duration of hospitalization due to COVID-19	73	136	0.0025
Days¶	12.1 (4.0)	13.9 (4.3)	
Patients discharged up to day 11#	39 (53.4)	39 (28.5)	0.0004
Patients discharged up to day 14#	59 (80.8)	90 (65.7)	0.0215
Patients requiring remdesivir therapy#	12 (16.4)	63 (46.0)	0.0001
Patients requiring corticosteroid therapy#	5 (6.8)	9 (6.6)	0.9383
Patients with all-cause mortality#	0	0	−

COVID-19 = coronavirus disease 2019; SpO_2_ = peripheral capillary oxygen saturation.

⁎ Primary end point: Fisher exact test.

† Secondary end points: χ^2^ test for categorical variables and Student *t* test for continuous variables.

‡ Values are presented as n (%) [95% CI]. Wilson 95% CI for each proportion.

§ Values are presented as n [95% CI]. Farrington-Manning score exact 95% CI for the proportional difference between cohorts.

|| Defined as body temperature maintained <38°C. Fever recovery duration was defined as [(first fever recovery date/time after last body temperature ≥38°C) – (first date/time of body temperature ≥38°C)].

¶ Values are presented as mean (SD).

# Values are presented as n (%).

### Safety profile

There were 2 patients with treatment-emergent AEs that were determined to be adverse drug reactions up to Day 28 after initial hospitalization. One patient had pruritus and rash and the other patient had pruritus. The severity of all the events was grade 1 and all resolved (see Table 6 for details). There were no serious AEs or discontinuations due to treatment-emergent AEs.

**Table 6:** Listing of adverse events in the regdanvimab cohort up to day 28.

Age, year Sex	System organ class/preferred term	Duration, d*	Treatment-emergent adverse event	Relationship to regdanvimab	Outcome	Intensity	Serious adverse event
42/Male	Skin and subcutaneous tissue disorders/pruritus	1	Yes	Possible	Resolved	Grade 1	No
42/Male	Skin and subcutaneous tissue disorders/rash	1	Yes	Possible	Resolved	Grade 1	No
51/Male	Skin and subcutaneous tissue disorders/pruritus	1	Yes	Possible	Resolved	Grade 1	No

⁎ Adverse event duration was calculated as (adverse event stop date – adverse event start date + 1).

## Discussion

This retrospective, observational cohort study indicates that the anti–SARS-CoV-2 mAb regdanvimab can prevent deterioration of COVID-19 in patients with mild-to-moderate disease. A statistically significantly lower proportion of regdanvimab-treated patients deteriorated with SpO_2_ <94% on room air up to day 28 compared with patients in the nonregdanvimab cohort. A statistically significant difference favoring regdanvimab over nonregdanvimab SoC was also observed for most of the secondary outcomes, including time to recovery of fever, proportion of patients with SpO_2_ <90% on room air, proportion of patients requiring supplemental oxygen and duration of supplemental oxygen, proportion of patients receiving remdesivir, duration of hospitalization, and proportions of patients discharged within 11 and 14 days. No patients died or required mechanical ventilation. In the subgroup of patients aged ≥60 years, there was a statistically significant difference between cohorts for the primary end point and most secondary efficacy end points. Regdanvimab was well tolerated and associated with few adverse drug reactions, and none that required discontinuation of therapy.

Several mAbs, including regdanvimab, have now received emergency use authorizations or full regulatory approval in various countries around the world, including the Republic of Korea, for the treatment of patients with mild-to-moderate COVID-19 symptoms.^13^^,^^23^ Observational studies of real-world data are vital for effective population health management and regulatory decision making, helping to address knowledge gaps that cannot be filled by clinical studies, and ultimately ensuring public acceptance of these new products. The primary finding of this study was that 26.1% fewer patients receiving regdanvimab deteriorated with SpO_2_ <94% on room air up to day 28 compared with patients receiving SoC (*P<*0.0001).

Low levels of oxygen saturation are known to be associated with poor prognosis and are a risk factor for mortality in patients with COVID-19.^18^^,^^19^ As such, pulse oximetry monitoring is recommended to monitor the clinical status of patients with a target SpO_2_ of 92% to 96%.^23^ Severe COVID-19 typically manifests about 1 week after the onset of symptoms, but some patients can be hypoxemic and be at risk of serious complications without symptoms of dyspnea.^23^^,^^24^ In the present study, median (range) SpO_2_ levels were 97.0% (92%–100%) at baseline, with no differences between cohorts. Regdanvimab reduced the requirement for supplemental oxygen by maintaining oxygen saturation levels ≥94%. In the SoC group, the rate of deterioration increased until Day 6, with more patients deteriorating to SpO2 <94% over this time. By contrast, almost no new cases of low oxygen saturation occurred after 3 days in the regdanvimab group, and the effect of regdanvimab was remarkable after Day 7. Given that patients were admitted on day 1 of the study (and assuming that hospitalization occurred approximately 3 days after symptom onset), this finding is consistent with the natural course of COVID-19.^25^ Moreover, the proportion of patients with SpO_2_ <90%—which is considered a medical emergency requiring oxygen therapy regardless of physical signs^26^—was 3 times higher in the nonregdanvimab cohort than in the regdanvimab cohort. Accordingly, there were significantly more patients with SpO_2_ low enough to warrant supplemental oxygen therapy in the nonregdanvimab cohort (52%) than in the regdanvimab cohort (24%), and the duration of supplemental oxygen therapy was shorter in the regdanvimab cohort (6.3 vs 8.7 days; *P =* 0.0113). No patients required mechanical ventilation. Approximately 80% of patients in the present study had moderate disease at baseline, as defined by the presence of pneumonia. As pneumonia is associated with poor prognosis in patients with mild to moderate COVID-19,^27^ and patients with moderate disease are more likely to progress to severe disease than those with mild disease,^28^ the low proportion of regdanvimab-treated patients who deteriorated in the present study is particularly striking.

Early use of mAbs appears to have an impact on both mortality and hospitalization. Real-world data in 246 elderly long-term care facility residents with mild-to-moderate COVID-19 showed significantly reduced mortality among those receiving bamlanivimab, in comparison with those who did not (3% vs 10%; odds ratio = 0.25; *P =* 0.03), significantly shorter time to resolution of fever (1.98 vs 3.9 days; *P <* 0.0001), and a trend toward reduced hospitalization (4.37% vs 10.46%; odds ratio = 0.35; *P =* 0.08).^29^ Similarly, lower 30-day hospitalization rates were observed with bamlanivimab treatment than with no mAb therapy in a US retrospective case-control series of 403 high-risk ambulatory patients with COVID-19 (7.3% vs 20.0%, relative risk = 0.37; *P <* 0.001).^30^ In a large, retrospective cohort study involving 2820 high-risk outpatients with mild to moderate COVID-19 who were offered mAb therapy (bamlanivimab or casirivimab-imdevimab), the 28-day hospitalization rate was higher among those declining treatment than those accepting treatment (3.3% vs 2.0%; rate ratio = 0.62).^31^ Our study was conducted in the Republic of Korea where all patients with symptomatic COVID-19 are treated in hospital; thus, it was not possible to evaluate prevention of hospitalization. However, a clear role for regdanvimab in preventing hospitalization associated with COVID-19 was demonstrated in the Phase III study.^11^^,^^32^ In our study, the mean duration of hospital admission was significantly shortened in the regdanvimab versus nonregdanvimab cohort (11.1 vs 13.6 days; *P <* 0.0001) despite the minimum quarantine period of 10 days recommended by Korean medical guidelines. In addition, the number of patients discharged up to day 11 was higher in the regdanvimab than nonregdanvimab cohort (63.8% vs 31.6%; *P <* 0.0001). Therefore, our study confirms, in a real-world setting, the findings of the Phase III study that regdanvimab prevents progression to severe COVID-19, defined as hospitalization, need for oxygen therapy, or death.^11^^,^^12^

Regdanvimab was well tolerated in the present study, aligning with data from the Phase III clinical study, which showed regdanvimab to have a favorable safety profile in patients with mild-to-moderate COVID-19, with no clinically meaningful differences in AE profile versus placebo.^11^ In the Phase III study, infusion-related reactions were mild and transient, with most patients recovering within 1 to 3 days.

This study has several limitations. The retrospective single-center design carries an inherent risk of bias and confounds the generalizability of the results to other institutions where different criteria might be applied to regdanvimab use. The number of patients in the 2 groups was not balanced as this was a retrospective study that analyzed the data of hospitalized patients during the specified period, not randomized trial. The nonregdanvimab cohort also received a range of treatments, and included slightly more elderly patients, confounding comparison between the 2 cohorts. Furthermore, data are only reported up to 28 days, limiting assessment of the long-term safety profile of regdanvimab, and may not fully cover the full clearance period for regdanvimab, which has a t_½_ of ∼12 days.^13^ Nevertheless, our real-world data provide an insight into the clinical efficacy of regdanvimab in patients with mild-to-moderate COVID-19 by using an easily measured outcome, and provide confirmation of its clinical efficacy and safety profile, which were in line with the clinical results reported in a Phase III clinical study.^11^

Although our data offer no insight into the clinical efficacy of regdanvimab against specific SARS-CoV-2 variants, animal models have shown therapeutic doses of regdanvimab to have in vivo neutralizing potency against B.1.351, suggesting that regdanvimab may have therapeutic potential in patients with COVID-19 who have been infected with the beta (South African) variant of concern,^33^ as well as the gamma, delta, epsilon, and kappa variants.^34^ Data are not yet available for the omicron variant.

## Conclusions

This retrospective analysis suggests that regdanvimab is clinically effective and prevents oxygen saturation deterioration in the real world among patients with mild COVID-19 at high risk of progression, and moderate COVID-19. No new safety issues were identified. These data provide further support of the potential benefits of regdanvimab, which adds to a limited list of treatments available for patients with mild COVID-19 at high risk of progression, and moderate COVID-19.
